# Performance of objective functions and optimisation procedures for parameter estimation in system biology models

**DOI:** 10.1038/s41540-017-0023-2

**Published:** 2017-08-08

**Authors:** Andrea Degasperi, Dirk Fey, Boris N. Kholodenko

**Affiliations:** 1 Systems Biology Ireland, University College Dublin, Belfield, Dublin, Ireland; 2 Wellcome Trust Sanger Institute, Wellcome Trust Genome Campus, Hinxton, Cambridge, UK; 3 School of Medicine and Medical Science, University College Dublin, Belfield, Dublin, Ireland; 4 Conway Institute of Biomolecular & Biomedical Research, University College Dublin, Belfield, Dublin, Ireland

## Abstract

Mathematical modelling of signalling pathways aids experimental investigation in system and synthetic biology. Ever increasing data availability prompts the development of large dynamic models with numerous parameters. In this paper, we investigate how the number of unknown parameters affects the convergence of three frequently used optimisation algorithms and four objective functions. We compare objective functions that use data-driven normalisation of the simulations with those that use scaling factors. The data-driven normalisation of the simulation approach implies that simulations are normalised in the same way as the data, making both directly comparable. The scaling factor approach, which is commonly used for parameter estimation in dynamic systems, introduces scaling factors that multiply the simulations to convert them to the scale of the data. Here we show that the scaling factor approach increases, compared to data-driven normalisation of the simulations, the degree of practical non-identifiability, defined as the number of directions in the parameter space, along which parameters are not identifiable. Further, the results indicate that data-driven normalisation of the simulations greatly improve the speed of convergence of all tested algorithms when the overall number of unknown parameters is relatively large (74 parameters in our test problems). Data-driven normalisation of the simulations also markedly improve the performance of the non-gradient-based algorithm tested even when the number of unknown parameters is relatively small (10 parameters in our test problems). As the models and the unknown parameters increase in size, the data-driven normalisation of the simulation approach can be the preferred option, because it does not aggravate non-identifiability and allows for obtaining parameter estimates in a reasonable amount of time.

## Systems biology: Performance of parameter estimation procedures

A systematic comparison of critical choices for faithful parameter-estimation identifies a combination of a hybrid optimisation algorithm (GLSDC) with data-driven normalisation of simulations (DNS) as the generally best option. Experimental data are often provided in relative, arbitrary units. To match simulations to data, two approaches are common: i) using scaling-factors that have to be estimated (SF); or ii) normalising the simulations in the same way as the data (DNS). Using three test-models of increasing complexity, we explored how this choice affects parameter identifiability and estimation performance. We show that in contrast to SF, DNS does not aggravate non-identifiability and a global-hybrid method combined with DNS outperformed local-multi-start methods. The advantage of DNS in terms of estimation speed was particularly pronounced for the most complex test-problem.

## Introduction

Signalling pathways constitute the machinery that cells use to sense, process and transmit information within and between cells. Because of the non-linear nature and the complexity of signalling pathways, mathematical modelling has been used to formalise the current understanding, identify inconsistencies and suggest new hypotheses. Dynamic models, such as ordinary differential equations (ODEs), are among widely used modelling approaches, capturing the quantitative and dynamic nature of cellular signalling pathways.^1, 2^ Mathematically, ODE models are of the form

1 $$\frac{{\rm{d}}}{{{\rm{d}}t}}x = f\left( {x,\theta } \right),$$

where $f\left( \cdot \right)$ is a nonlinear function of state vector *x*. The ODE describes how the rate of change d*x*/d*t* depends on *x* and kinetic parameters *θ*. An ODE solution is a function (*x* = *x*(*t*,*θ*)) that depends on time and parameters. Successful examples of dynamic modelling include elucidating the decision-making mechanisms in growth factor signalling,^3, 4^ stress and DNA damage response^5, 6^ and cell migration.^7^

With ever-increasing pace of documenting molecular interactions within and between signalling pathways, there is a need to develop larger and more complex mathematical models. While our current knowledge of molecular interactions allows us to derive kinetic equations of a model in a relatively straightforward manner, the associated increase in unknown kinetic parameters presents a challenge. Usually, these parameters are not directly experimentally accessible. Parameter estimation is the process of indirectly estimating the unknown parameter values using measurement data, which requires high-resolution data of time-courses and multiple perturbations.^4, 8, 9^ The complexity and non-linearity of biological systems render the parameter estimation problem mathematically difficult. Issues arise from both the existence of local minima and non-identifiability. To overcome local minima, heuristic optimisation-based algorithms are used.^10, 11^ Non-identifiability means that a unique solution to the parameter estimation problem does not exist. Thus, there are many sets of parameter values that fit the data equally well. Non-identifiability can only be overcome by model reformulation or model reduction,^12^ or by generating additional data, for example by measuring additional variables.^13^

Usually only a subset of all the internal states (or a function thereof) are measured, referred to as observables $\tilde y$.^9, 14^ To describe the relation between the states and observables in the model, a so-called output function is used *y* = *g*(*x*). Based on the simulated (*y*) and measured ($\tilde y$) observables, a parameter estimation problem can be formulated as an optimisation problem, in which the error between measured and simulated values is minimised.^9^ Mathematically, this error is described using an objective function (sometimes also called the goodness-of-fit function). Several choices for such objective functions exist. Most common are least-squares (LS), chi-square and log-likelihood (LL).^15^

A multitude of optimisation algorithms exists that estimate parameters of dynamic models.^16^ A recent comparison found that LSQNONLIN SE (a local gradient-based search algorithm with Latin hypercube restarts) performs best in terms of both accuracy and speed (as measured in the number of function evaluations required to estimate the parameters).^17^ In particular, this algorithm largely outperformed 14 other algorithms used to solve non-linear optimisation problems, including stochastic algorithms from an Evolutionary Algorithms framework (EvA2)^18^ and a hybrid stochastic-deterministic algorithm based on scatter search.^19^ Thus, LSQNONLIN SE has become a popular choice for parameter estimation of systems biology models.^20–23^

It is reported that hybrid stochastic-deterministic methods perform better than local gradient-based methods with restarts for complex problems.^24, 25^ Hybrid methods combine stochastic strategies, which help to escape local minima, with deterministic local strategies, which quickly find local optimal solutions.^24, 25^ For instance, previously, we used GLSDC,^26^ a hybrid algorithm that was not included in the above comparison,^17^ that time-efficiently estimated parameters for a complex model of expression of the transcription factor cFOS.^3^ However, it is unknown how GLSDC compares to other algorithms, in particular LSQNONLIN SE, which presumably is the currently fastest choice according to ref. 17.

A practical problem arising for any parameter estimation is how to scale the simulated data points to the measured data. This is because the most common type of experimental data are relative data (e.g. western blotting,^27, 28^ multiplexed Elisa,^29^ proteomics or RT-qPCR^30^), which means that the values of the data points are in arbitrary units (au), such as optical densities.^31^ In contrast, mathematical models carry well-defined units, such as molar concentrations or normalised dimensionless variables.^9, 17, 24^ For example, quantification of a western blot image may yield density values for a time-course experiment between 10 and 3000 au, whereas the model simulates nano-molar concentrations between 0 and 500 nM. Consequently, the problem arises of how to best align the measured and simulated data. Two approaches are commonly used:

1. Introducing a scaling factor (SF) that scales simulated to measured data ${\tilde y_i} \approx {\alpha _j}{y_i}\left( \theta \right)$, where the “≈” sign indicates that the match is not perfect due to modelling and measurement errors, ${\tilde y_i}$ and *y*_*i*_ denote a measured and simulated data-point, respectively, and ${\alpha _j}  >0$ is the SF for this observable. The SF is unknown and has to be estimated.
2. Normalising simulations and data in the same way (data-driven normalisation of the simulations (DNSs)). Experimental data are often normalised by a reference data point to make different biological replicates comparable^31^ (${\tilde y_i} = {\hat y_i}/{\hat y_{{\rm{ref}}}}$, with ${\hat y_i}$ the un-normalised data). Then we can use the same normalisation for the simulated data, ${\tilde y_i} \approx {y_i}/{y_{{\rm{ref}}}}$. The reference data point *y*_ref_ could for example be the maximum value, the control, or the average of all measured values for this observable in this replicate.

Note that DNS normalises the simulations, rather than experimental data. Such normalisation is not required for data-driven, machine-learning-type models that simulate the data directly in whatever unit these might be, but critical for the type of mechanistic models considered here that simulate biological processes rather than data. Further, even if the measured data are not normalised, we can use DNS by employing the same normalisation on both data and simulation.

Although DNS has the advantage, with respect to SF, of not introducing additional unknown parameters, DNSs are rarely used. A reason can be that current parameter estimation software, such as COPASI^32^ and Data2Dynamics,^33^ do not include DNS support. A technical difficulty arises, because the normalisation has to be applied to each simulation run, after the run is completed. Unlike the SFs, the normalisation factors *y*_ref_ cannot be a-priori fixed, because they dynamically depend on the simulation. In principle, DNS could be incorporated into the objective function, but this would require encoding custom-specified objective functions for each parameter estimation problem (Supplementary Method 1). User-friendly software supporting DNS is lacking. Further, a rigorous, systematic comparison of using DNS vs. commonly used SF has never been performed.

Here, our main objective is to provide a software fully supporting DNS (PEPSSBI^34^) and establish how the choice of using either SF or DNS affects identifiability and estimation convergence-speed in the context of different parameter optimisation algorithms and objective functions. We analysed three test-bed parameter estimation problems with markedly different numbers of observables (one or eight) and unknown parameters (10 or 74). Our results show that (i) accurately assessing the convergence speed of algorithms employing sensitivity equations (SEs) requires measuring the computation time: counting function evaluations is inappropriate in this setting; (ii) unlike SF, using DNS does not aggravate non-identifiability problems and improves optimisation performance in terms of speed compared to SF; (iii) For large parameter numbers, GLSDC performs better than LevMar SE, an implementation of the current best performing method (LSQNONLIN SE) in ref. 17.

## Results

### Objective functions, optimisation algorithms and test-bed problems

We focus on systematic comparison of DNSs vs. SFs for parameter estimation in dynamic systems. We consider least squares (LS) and log-likelihood (LL) objective functions (Supplementary Method 1) and three optimisation algorithms (Methods):

1. LevMar SE: Levenberg–Marquardt nonlinear least squares optimisation algorithm^35^ with SEs. LevMar SE uses gradient-based local optimisation with Latin hypercube restarts whereby the gradient is computed using SEs^17^;
2. LevMar FD: like LevMar SE except that the gradient is computed using finite differences (FDs), which we included for comparing the FD and SE approaches;
3. GLSDC: Genetic Local Search algorithm with distance independent Diversity Control, which alternates a global search phase based on a genetic algorithm with a local search phase based on Powell’s method and does not require computation of the gradient.^26^

LevMar SE and FD are our implementations of LSQNONLIN SE and LSQNONLIN FD studied in ref. 17 (Methods).

We analyse three parameter estimation problems as test problems, which for brevity we call STYX-1-10 (ref. 4), EGF/HRG-8-10 (ref. 3) and EGF/HRG-8-74 (ref. 3). In this notation, the first number indicates the number of observables (and thus SFs, if SF is used), and the second number indicates the number of unknown kinetic parameters. Two additional unknown parameters (*s*_*a*_ and *s*_*b*_, see Supplementary Method 1) are estimated if LL is used.

The STYX-1-10 problem consists of a dynamic model describing the interactions between the kinase ERK and the pseudophosphatase STYX following the activation of ERK kinase MEK in PC12 cells.^4^ Data for one observable, phosphorylated ERK, in two experimental conditions, control and STYX knock down, are available. Overall, the model consists of 25 species, 22 reactions and 42 parameters, 10 of which require estimation. The experimental data contained 38 data points, and normalisation by average was used to normalise the data. For details about relative data normalisation see Supplementary Method 1 and ref. 31.

The EGF/HRG-8-10 and EGF/HRG-8-74 problems consist of a mathematical model of the ERK pathway activation and two transcriptional negative feedback loops triggered by the activation of ERK kinase MEK in MCF-7 cells.^3^ Data for eight observables in two experimental conditions, EGF or HRG stimulations, are available. Overall, the model consists of 49 species, 78 reactions and 141 parameters. In EGF/HRG-8-74, 74 parameters require estimation, while in EGF/HRG-8-10, only 10 parameters require estimation, and the remaining 64 parameters are assigned values from the best optimal parameter set estimated in the original publication.^3^ The data consist of 112 data points, and normalisation by average was used to normalise the data.

### The test problems exhibit different degrees of parameter non-identifiability

**Fig. 1:**
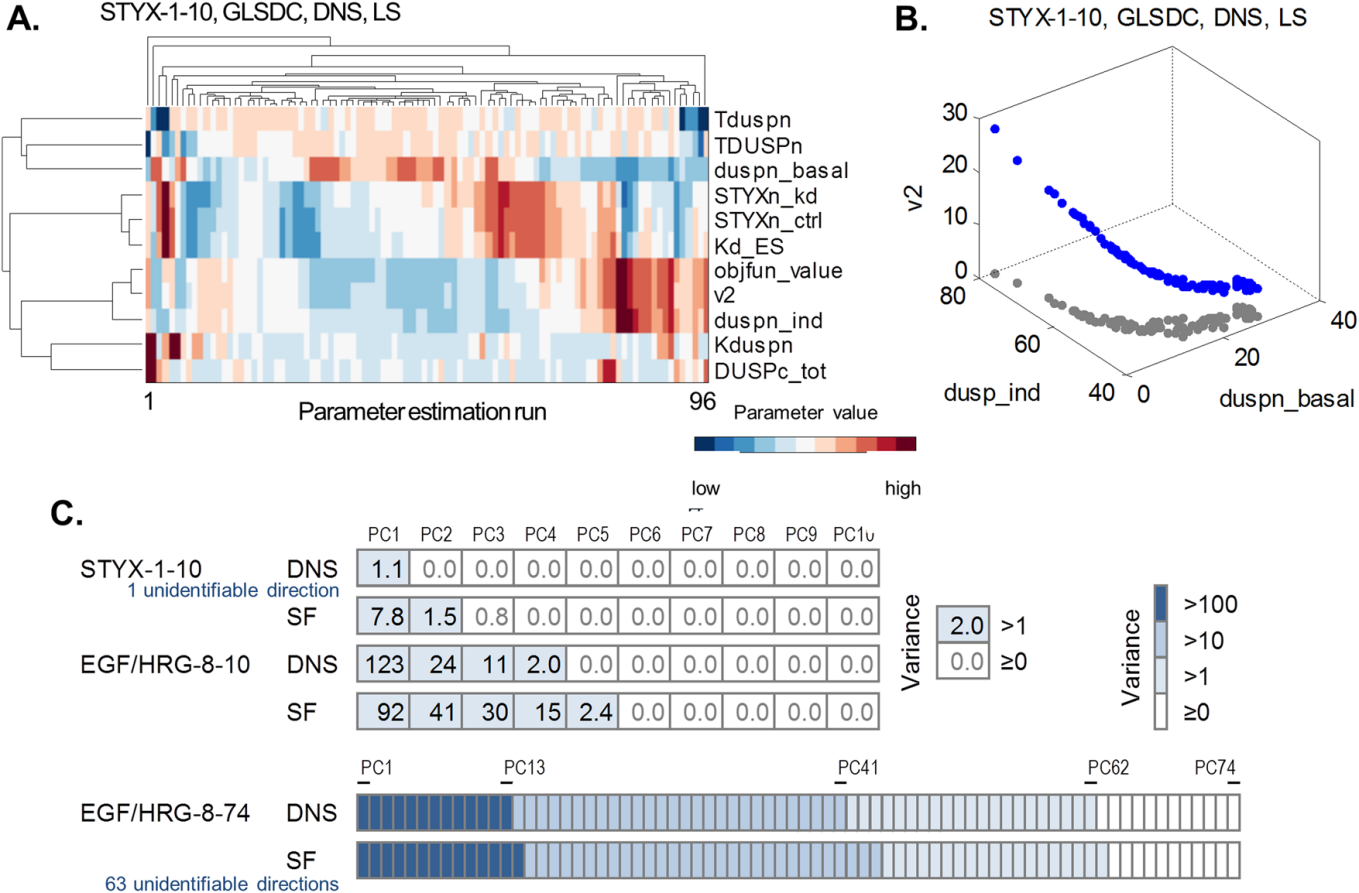
Relations between parameter estimates and non-identifiability. **a** Clustergram visualising the relations between the parameter estimates. **b** Scatter plot illustrating that the space occupied by the estimates is a low-dimensional manifold: here a 1D curve in 10D space; shown is a projection in 3D (*blue dots*), and 2D (*grey dots*). **c** Principal component analysis of the parameter estimates. *Bars* illustrate the number of principal components required to explain the variability of the parameter estimates. The *colours* indicate how much variance is explained by each principal component (*PC*). The data were brought onto the same scale by normalising each parameter with respect to the best estimate from GLSDC-DNS-LS (see Methods). The number of PCs required indicates the dimensionality of the estimated parameter space (linear approximation), thus indicating the degree of non-identifiability

Performance of different parameter estimation schemes may critically depend on the identifiability properties of test problems. Therefore, we analysed practical (a posteriori) identifiability.^36^ Briefly, by analysing the parameter estimates from multiple runs, we assess practical non-identifiability. If the parameter estimates are largely variable in a certain direction, then this direction in the parameter space (e.g. a particular parameter or a parameter combination) is not practically identifiable: their parameter values cannot be accurately estimated given the current model and data. None of our test problems was practically identifiable (Fig. 1). Nevertheless, each problem exhibited different degrees of non-identifiability, with STYX-1-10 having the lowest degree of non-identifiability, and EGF/HRB-8-74 the highest. Here we define the degree of practical (a posteriori) non-identifiability as the number of directions in the parameter space that are not identifiable. This is best explained using an example.

The parameter estimates for the STYX-1-10 problem were highly related, where all estimates clustered along a one-dimensional (1D) curve in the high-dimensional parameter space (Fig. 1). Parameter changes along this curve are not identifiable, because they lead to very similar objective function values. All other directions are identifiable, because moving away from the curve yields increased objective function values. Thus, the degree of non-identifiability of the STYX-1-10 DNS SF test problem is one. For parameter estimates that resemble a 1D curve, there is only one uncertain direction in the parameter space (along this curve); the degree of non-identifiability is one. Similarly, if the manifold of the estimated parameters resembles a 2D surface, there would be two uncertain directions (Supplementary Fig. 1), and the degree of non-identifiability is two.

Generally, a degree of practical non-identifiability is difficult to illustrate, because accurate visualisation of the estimated parameter space is not possible beyond three dimensions (three parameters). However, using a linear approximation, the dimensionality of the non-identifiable manifold can be estimated using principal component analysis (PCA). For details, we refer to Gutenkunst et al.^36, 37^. Briefly, PCA gives optimal directions in the parameter space, so-called principal components (PCs) that can best explain variability of the parameter estimates. PCs are ordered according to their associated variances, with the first PC explaining the most variability. Here, we considered a PC significant if its variance is >1, corresponding to more than a one-fold change with respect to the best estimate found, see Methods for details. Figure 1c shows that our test problems have different degrees of non-identifiability. STYX-1-10 has one non-identifiable direction, EGF/HRG-8-10 has four and EGF/HRG-8-74 has 62.

### Using SF over DNS increases non-identifiability because of the additional scaling parameters to be estimated

SFs constitute additional parameters to be estimated. Thus, we would expect that introducing these SFs negatively affects the identifiability of our test problems. Our a posteriori non-identifiability analysis using PCA confirmed this. Note that we focused the PCA analysis solely on the free kinetic parameters. The SF estimates and their variabilities were neglected, because we were only interested in the identifiability of the kinetic parameters (see Methods for details). Compared to DNS, using SF leads to one more non-identifiable PC for the STYX-1-10 and EGF/HG-8-10 test problems, and three more non-identifiable PCs for the EGF/HRG-8-74 test problem (Fig. 1). Thus, using SF (instead of DNS) increased the degree of non-identifiability in all our test problems.

We can have a closer look at the STYX-1-10 example to see what exactly happens. When DNSs are used, all estimates lie closely around a 1D curve in the parameter space. But when SFs are used, a distinct additional cluster occurs (Supplementary Fig. 1). The cluster is far away from the 1D curve (and the first PC), and represents another ‘pocket’ of parameter values with similarly low objective function values. In the DNS-LS case, this cluster contains 39 of the 96 estimates. Thus, there are two non-identifiable regions in the parameter space when SFs are used; the 1D curve also present in the DNS case, and the “pocket” (second cluster) introduced by the SFs. Further, when SFs were used, many kinetic parameters tended to cluster together with a SF, instead of another kinetic parameter that is biologically or mechanistically related, as was the case when DNS was used (Supplementary Fig. 2). The example illustrates how introducing SFs increase the degree of non-identifiability.

### Comparing LevMar SE and LevMar FD by counting function evaluations is inaccurate

**Fig. 2:**
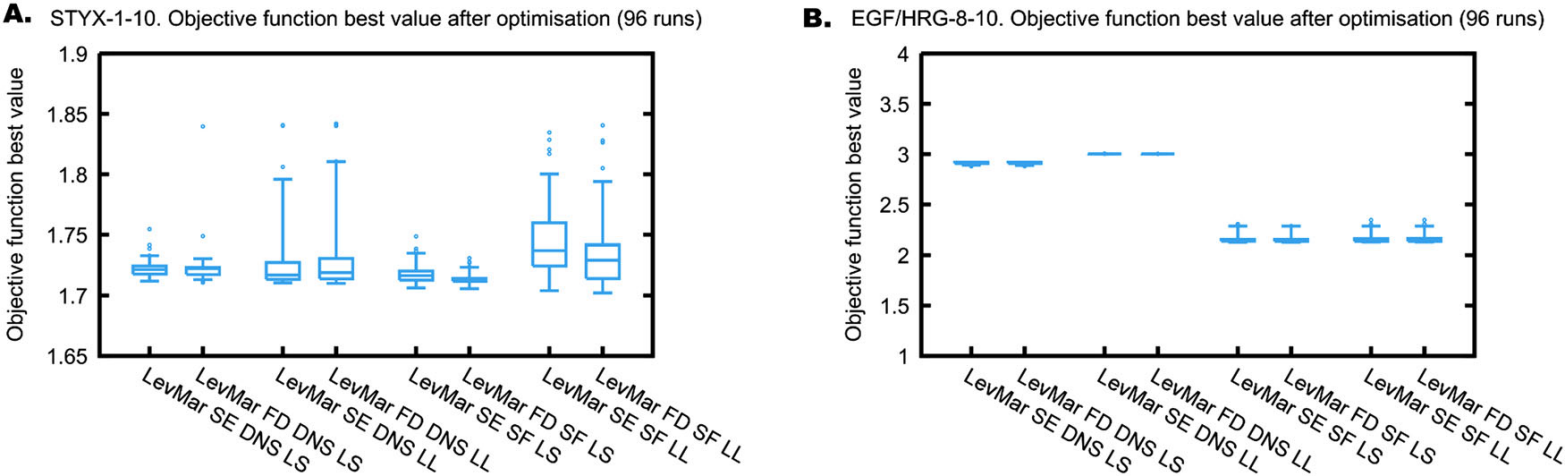
Minima reached by LevMar SE and LevMar FD after the optimisation terminated. *Boxplots* show the median, 25th and 75th percentile, and extreme points (*dots*) outside 1.5 times the interquartile range (*whiskers*) of 96 independent runs. **a** STYX-1-10 optimisation problem. **b** EGF/HRG-8-10 optimisation problem. *DNS* normalisation of the simulations, *LL* log-likelihood, *LS* least squares, *SF* scaling factors. To facilitate the comparison, we always report the log-likelihood values on the *y* axis, even when LL was optimised

**Fig. 3:**
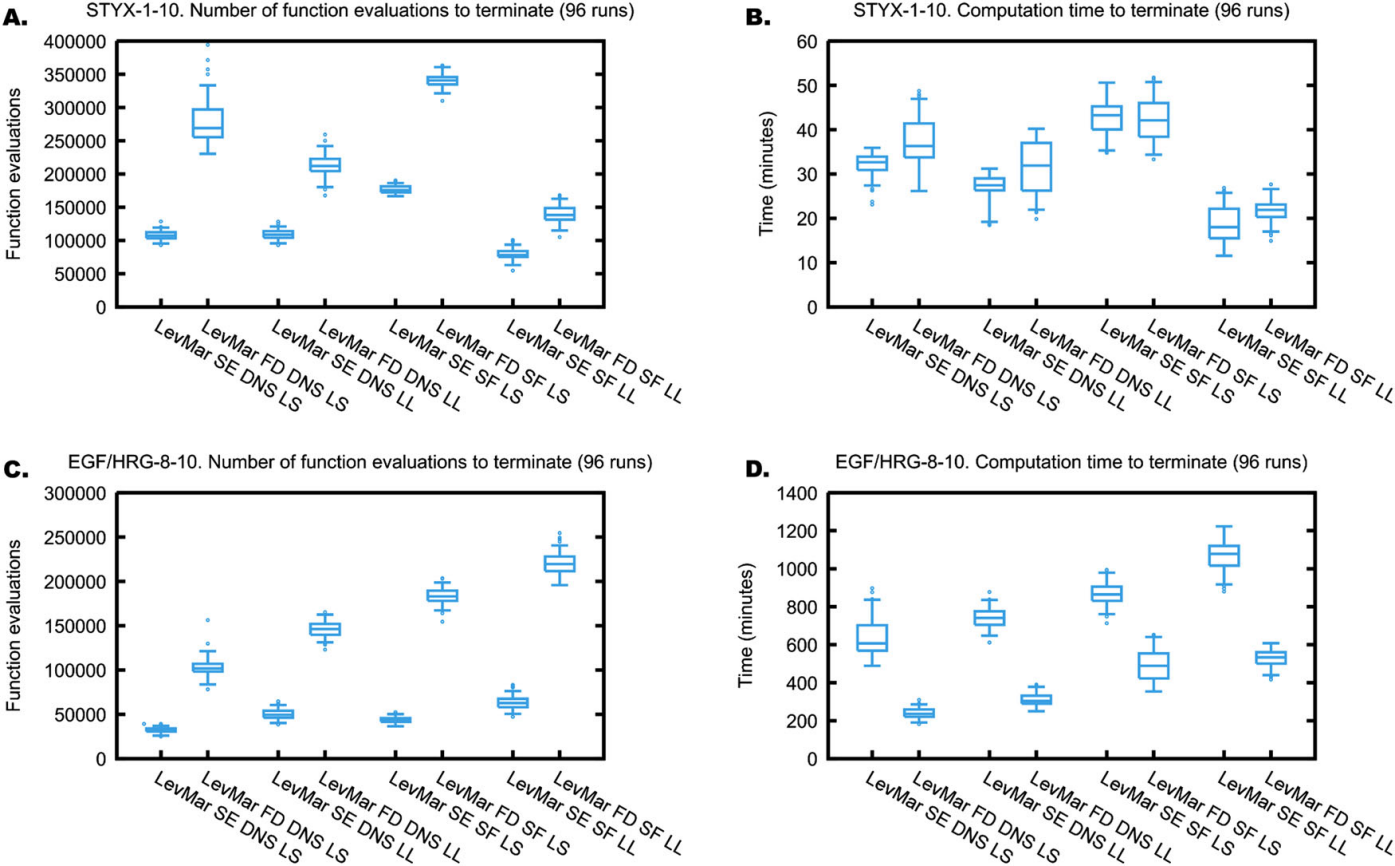
Convergence speed of LevMar SE and LevMar FD. Boxplots (*n* = 96) as in Fig. 1. **a** Required number of function evaluations to terminate and **b** computation time to terminate for the STYX-1-10 problem. **c** Required number of function evaluations to terminate and **d** computation time to terminate for the EGF/HRG-8-10 problem. *DNS* normalisation of the simulations, *LL* log-likelihood, *LS* least squares, *SF* scaling factors

Counting function evaluations in-lieu of actual computation times can lead to unfair comparisons. To illustrate this, we compare LevMar SE and LevMar FD using two test problems: STYX-1-10 and EGF/HRG-8-10 (Figs. 2 and 3). First, we have to make sure that both algorithms converge. For both test problems, the distribution of the optimal objective function values after termination of the algorithm shows no marked differences between LevMar SE and LevMar FD (Fig. 2). We conclude that both algorithms converge to equally good solutions.

Because LevMar FD and LevMar SE reach the same objective function minima and have the same termination criteria, we can compare the two algorithms by analysing the function evaluations to terminate optimisation or the termination time. This is illustrated in Fig. 3. For both test problems, we observe that using the number of function evaluations until termination as a performance measure leads to an overestimation of LevMar SE performance when compared to LevMar FD. This is because the count of function evaluations does not accurately reflect the actual computation time necessary to terminate. For both STYX-1-10 and EGF/HRG-8-10, the number of function evaluations to terminate is markedly lower for LevMar SE than for LevMar FD (Fig. 3a, c). Yet, the actual computation time only shows marginal improvements in the case of STYX-1-10 (Fig. 3b). Strikingly, in the case of EGF/HRG-8-10, the computation time is markedly and consistently higher for LevMar SE than for LevMar FD (Fig. 3d).

This apparent inconsistency is explained by the fact that for LevMar SE, the computational cost of computing the gradient is not reflected appropriately by the count of function evaluations. The gradient is either computed as a forward finite difference approximation in the case of LevMar FD, or supplied by solving the SEs in the case of LevMar SE. LevMar FD will approximate the gradient by computing *p* + 1 evaluations of the objective function, where *p* is the number of unknown parameters, solving only the original ODE system each time. LevMar SE evaluates the gradient by solving an ODE system that has *p* + 1 times the number of equations of the original ODE system, as it includes the original ODE variables and their derivatives with respect to the unknown parameters.^17^ Note that the cost of computing the SEs is much greater than the cost of simply solving the original ODEs. The cost of computing the gradient using SEs may be lesser or greater than the cost of computing the gradient using finite differences, depending on both the implementation and the specific optimisation problem. However, in both implementations of LevMar SE (here) and of LSQNONLIN SE in Data2Dynamics,^33^ computing the gradient is counted only as one function evaluation, whereas computing it with FD is counted as *p* + 1 function evaluations. Figure 3 demonstrates that this way of counting is not appropriate when comparing LevMar FD with LevMar SE, and that the actual computation time should be used instead.

### LevMar FD can be faster than LevMar SE

It has been argued that solving the SEs leads to a more accurate gradient than using a finite difference approximation, which in turn results in a faster converging algorithm in the SE case.^17^ In contrast, in our implementation LevMar SE is not always faster than LevMar FD. In fact, the case of EGF/HRG-8-10 shows the opposite; the computation time is always markedly lower for LevMar FD than for LevMar SE (Fig. 3d). How the computation time compares between LevMar SE and LevMar FD depends on both accuracy and cost of the gradient computation. At least for the EGF/HRG-8-10 test problem, our implementation of LevMar FD provides sufficient accuracy and converges faster than SE.

### DNS converges faster than SF, especially for systems with many observables, independently of optimisation algorithms used

One of the most important differences between DNS and SF is that SF requires the estimation of additional parameters: the SFs. For example, EGF/HRG-8-10 problem features 8 observables and 10 unknown kinetic parameters. Using DNS, only the 10 kinetic parameters need to be estimated. But using SF introduces 8 additional parameters, 1 unknown SF for each observable, thus 10 + 8 parameters need to be estimated. One would expect that this increase in the parameter numbers negatively affects the convergence speed of the optimisation algorithms, in addition to increasing the degree of parameter non-identifiability. This negative effect could become more pronounced, the more parameters require estimation. To determine whether these hypotheses are correct, we performed parameter estimations in three test problems that differ in terms of their numbers of observables and unknown parameters (STYX-1-10, EGF/HRG-8-10 and EGF/HRG-8-74).

**Fig. 4:**
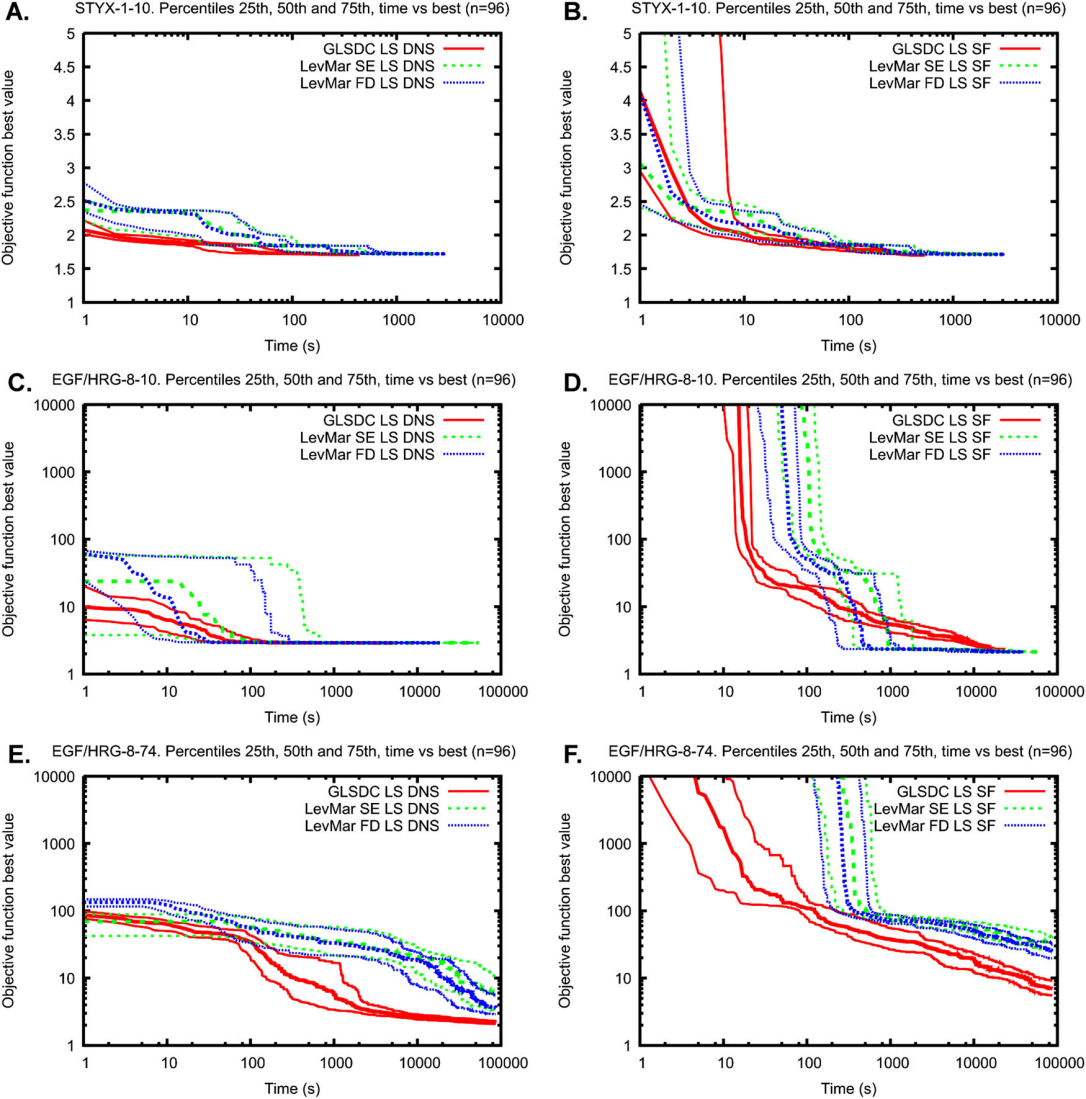
Convergence of the three optimisation algorithms for **a**, **b** the STYX-1-10 problem, **c**, **d** the EGF/HRG-8-10 problem and **e**, **f** the EGF/HRG-8-74 problem. The plots show the median (*thick line*), and 25th and 75th percentiles (*thin lines*) of the objective function minima (least squares values) over the computation time from independent 96 runs. **a**, **c**, **e** Normalisation of the simulations (*DNSs*). **b**, **d**, **f** Scaling factors (*SFs*)

**Fig. 5:**
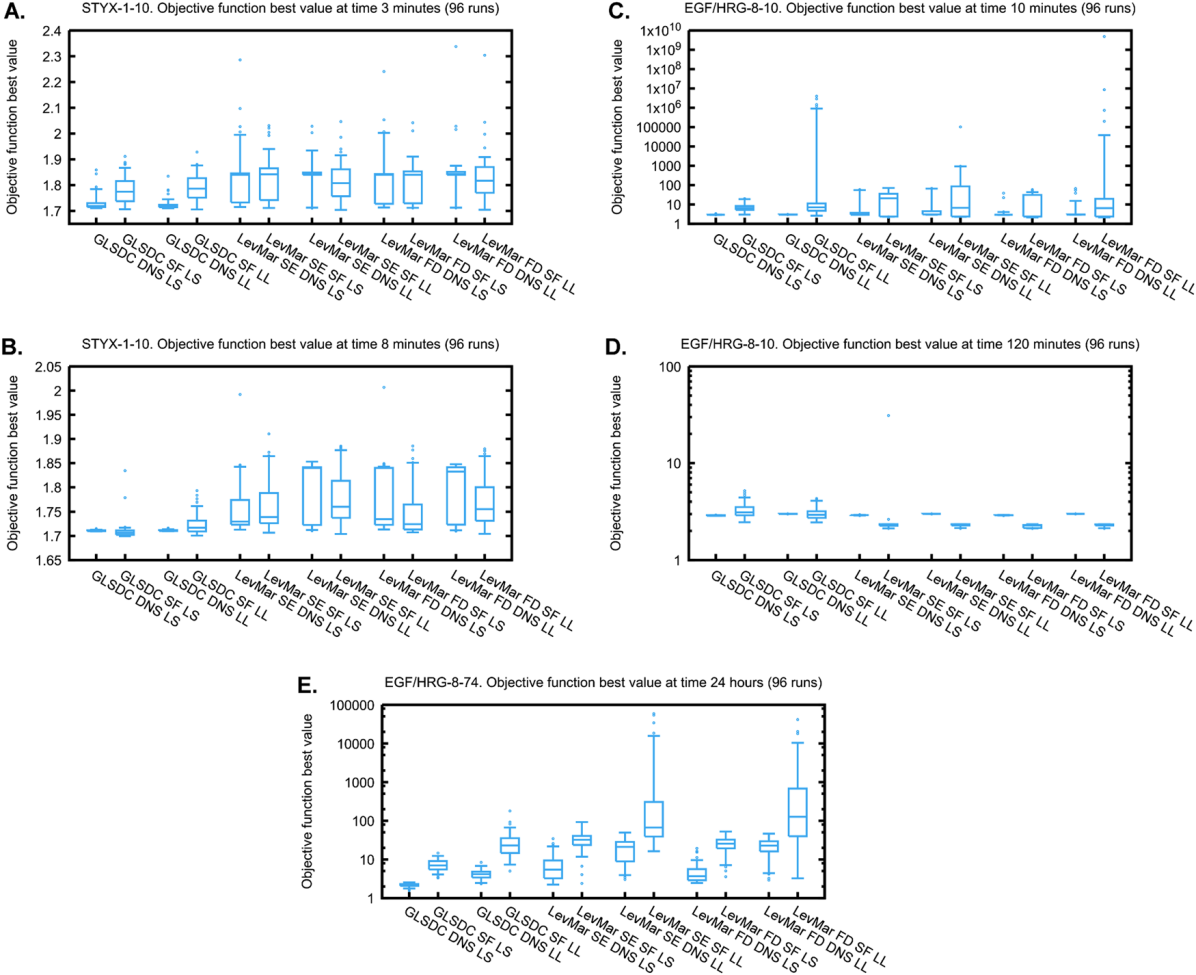
Minima reached by different algorithm-objective function combinations after a set time for **a**, **b** the STYX-1-10 problem, **c**, **d** the EGF/HRG-8-10 problem and **e** the EGF/HRG-8-74 problem. Times are **a** after 3 min, **b** after 8 min, **c** after 10 min, **d** after 2 h and **e** after 24 h of optimising. Boxplots as in Fig 1. *DNS* data-driven normalisation of the simulations, *LL* log-likelihood, *LS* least squares, *SF* scaling factors. To facilitate the comparison, we always report the LS values on the *y* axis, even when LL was optimised

For the STYX-1-10 problem with only one observable, using DNS instead of SF improved the convergence speed of GLSDC for both LS and LL objective functions, while the same improvement was not observed for LevMar (Figs. 4a, b and 5a, b). For the more complex problems, all tested algorithms converged faster when DNS was used instead of SF: albeit the convergence speed increased only slightly for the EGF/HRG-8-10 problem featuring more observables but the same number of unknown kinetic parameters as in the STYX-1-10 problem, this increase was very substantial for the EGF/HRG-8-74 problem featuring both more observables and more parameters (Figs. 4 and 5). Both observations were independent of the algorithm (GLSDC, LevMar SE, LevMar FD) and objective function (LS, LL) used. Thus, choosing DNS over SF consistently improved the convergence speed of the estimation. This gain in performance became more pronounced for more complex problems with more observables (8 vs. 1) and more parameters (74 vs. 10).

In addition to the SFs introduced by SF, using LL also introduces additional parameters. The LL objective function features an error model for estimating the variances for each measured data-point (Supplementary Method 1). This error model contains two parameters that need to be estimated relating to the absolute and proportional part of the measurement error. As a consequence, the convergence speed of the most complex test problem (EGF/HRG-8-74) was markedly reduced when LL was used instead of LS (Fig. 5e). For this complex problem, estimating both eight additional scaling parameters (DNS vs. SF) and the two additional error parameters (LS vs. LL) negatively affected the convergence of all three algorithms tested.

### GLSDC DNS was the fastest in all test problems, performing particularly well for problems with many parameters

Recently, LSQNONLIN SE, an alternative implementation of LevMar SE, was reported as the best performing algorithm in a comprehensive test including 15 algorithms and 2 test problems.^17^ But neither the GLSDC algorithm, nor simulation scaling with DNS, were part of this comparison, raising the question of whether GLSDC could outperform LevMar SE. We found that when combined with DNS, GLSDC was consistently the fastest in all our test problems (Fig. 5a, c, e). The performance of GLSDC critically depended on the use of DNS or SF: for the EGF/HRG-8-10 problem, GLSDC was the fastest converging algorithm when DNS was used, while it was among the slowest when SF was used (Fig. 5c, d). This is particularly evident when looking at the full convergence curve (Fig. 4c, d). Similarly, GLSDC with DNS was also the fastest for the EGF/HRG-8-74 problem. Albeit a decrease in performance occurred when SF was used, GLSDC SF was still markedly faster compared to the LevMar SF combinations. In fact, for this high-dimensional problem, the performance increase of GLSDC over LevMar was quite dramatic (Fig. 4e, f). As a result, GLSDC DNS LS stands out as the only algorithm-objective function combination capable of reaching acceptable parameter estimates robustly within a reasonable timeframe for complex systems (24 h, 8 observables, 74 unknown parameters in our test problem).

## Discussion

Here we analysed how the overall performance of a parameter estimation procedure depends on optimisation algorithms, the selected objective functions (e.g. LS vs. LL), and the scaling/normalisation used (SF vs. DNS). When making such comparisons, choosing an appropriate performance metric is important. In particular, we demonstrated that when comparing LevMar SE with LevMar FD, counting of the function evaluations overestimates the performance of SE. When gradient-based methods are used, the computational cost of calculating the gradient cannot be neglected. Thus, the actual computation time has to be used.

First, we analysed how the algorithms perform depending on the number of SFs and number of unknown parameters. In high-dimensional problems with several SFs (EGF/HRG-8-74, 8 observables, 74 parameters), GLSDC is the best performing algorithm (Figs. 4f and 5e). In low-dimensional problems with one SF (STYX-1-10, one observable, ten parameters), the three algorithms have similar performances (Fig. 4b). In low-dimensional problems with several SFs (EGF/HRG-8-10, eight observables, ten parameters), LevMar FD and SE have an overall better convergence than GLSDC (Figs. 4d and 5d). The results are consistent with previous results, in which hybrid methods (enhanced scatter searches) outperformed gradient-based methods with hypercube restarts.^24^ The fact that the LSQNONLIN SE outperformed the hybrid stochastic methods in ref. 17 may be due to the use of function evaluations as performance measure. A systematic comparison of GLSDC with the best performing hybrid method in ref. 17 and the novel hybrid method in ref. 24 is currently lacking, and can be the subject of a future study.

Next we showed how the choice between DNS and SF affects both identifiability and convergence speed of the optimisation algorithms. For problems with several SFs (EGF/HRG-8-10 and EGF/HRG-8-74), using DNS improves the performance of every algorithm tested (Figs. 4c–f and 5c–e). For problems with a few SFs (STYX-1-10), using DNS markedly improves the performance of GLSDC but not LevMar FD and SE (Figs. 4a, b and 5a, b). The results are consistent with the notion that gradient-based methods are more efficient in relatively small problems that tend to be well behaved (only one or two non-identifiable parameter directions in the STYX-1-10 case), whereas hybrid methods perform better for complex problems with a high degree of non-identifiability.^11, 24, 38^ For all problems, DNS is either neutral or beneficial, and should thus be the first choice.

The fact that SF reaches lower minima while at the same time increasing the variability in the parameter estimates would indicate that the risk of overfitting is very high. Overfitting occurs when free parameters are used to fit noise rather than biologically meaningful trends. Although the risk of overfitting is generally higher when more parameters are fitted, the structure of the dynamic model also plays a key role:^24, 38^ flexible models that can exhibit a range of behaviours (for example due to multiple feedback loops^39, 40^) are more prone to overfitting even when the number of parameters remains low.^41, 42^ That the SFs might be used by the optimisation to overfit the data is also supported by our observation that many kinetic parameters clustered together with a SF, instead of another biologically related parameter. But whether DNS carries an advantage over SF in terms of overfitting is an open question that should be the subject of a follow-on study. For example, using only parts of the experimental data and bootstrapping could be employed in a systematic analysis of overfitting.

Finally, we have seen that as the number of unknown parameters increases, the benefit of using DNS over SF increases. In fact, in the STYX-1-10 case the choice of estimating SFs (DNS vs. SF) and estimating the measurement error (LS vs. LL) does not seem to affect the convergence of the gradient-based algorithms LSQNONLIN FD and SE (Fig. 5a, b). However, in the case of EGF/HRG-8-74 both these choices have a clear impact on convergence speed (Fig. 5e). Our results suggest that for such high-dimensional problems, GLSDC DNS stands out as the best performing algorithm in terms of convergence speed.

## Methods

### Software implementation

The analysis in this paper was performed in PEPSSBI (Parameter Estimation Pipeline for Systems and Synthetic Biology), a dedicated software that we developed and that is freely available at https://bitbucket.org/andreadega/systems-biology-compiler. This software provides an implementation of the three optimisation algorithms and the four objective functions tested here. Most importantly, PEPSSBI automates the set-up of the parameter estimation problems, including the ones based on the DNSs and automates the deployment of those problems to a computer cluster. A dedicated input language allows for model definition, data specification and normalisation in a single input file, which allows the user also to change data normalisation by changing a single line of code. When measuring computation time performance, we used a computer cluster with highly homogeneous nodes, composed of 64 Intel Ivybridge E5-2620 v2 and a 8 Intel Ivybridge E5-2620, which differ mostly on energy consumption and 4% clock speed.

### Sample size of the simulation runs

The sample size for all simulation runs is *n* = 96. This number was chosen as a compromise between statistical power, computation time and the cluster job submission specification (batches of 24 parallel jobs, to match the cluster hardware setup).

### Algorithm implementations

The GLSDC implementation was kindly provided by Shuhei Kimura (Tottori University, Japan). The LevMar SE algorithm was implemented using the levmar C library, which is an open source implementation of the Levenberg–Marquardt nonlinear least squares algorithm.^43^ In contrast, LSQNONLIN SE and SD in ref. 17 use the Matlab built-in function “lsqnonlin.m”. Similar to LSQNONLIN SE and SD, we used the CVODES (Sundials) C library^44^ to solve the ODEs and the SEs. More information about the algorithms and objective functions is available in Supplementary Method 1.

### Practical identifiability analysis using PCA

We used PCA to analyse a posteriori identifiability. For each parameter, we log-normalised the data (estimates) with respect to the best estimate from GLSDC-DNS-LS. *θ*_*i*,*j*,norm_ = log_2_(*θ*_*i*,*j*_/min_*j*_ *θ*_*i*,*j*_), where *i* indicates the parameter, and *j* the estimate. Thus, a value of 1 in the log-normalised data indicates a two-times change with respect to the best estimate. PCA of the log-normalised parameter data was performed using the princomp function in Matlab (R2010a), with parameters in the columns and estimation results (runs) in the rows. We considered a parameter direction unidentifiable, if the associated variance as revealed by PCA is >1.

*Remark*: Although related, this identifiability analysis is different from parameter sloppiness, which analyses how variable the directions are with respect to each other.^37^ If all parameters are variable, the system is not “sloppy”, yet it can be unidentifiable if the variance is large^36, 45^).

### Code and data availability

PEPSSBI is freely available at https://bitbucket.org/andreadega/systems-biology-compiler. Version 2.1 was used. The code implementing the test problems (including models and data) and scripts used to produce the results are available at https://bitbucket.org/andreadega/systems-biology-compiler/downloads/2017_03_30_PEPSSBI_performance_scripts.zip.

## Electronic supplementary material

Supplementary Methods 1

Supplementary Figure 1

Supplementary Figure 2
