# Supplementary material for: Performance of objective functions and optimisation procedures for parameter estimation in system biology models
Source: NPJ Syst Biol Appl. 2017 Aug 8;3:20. doi: 10.1038/s41540-017-0023-2 (PMC5548920; doi:10.1038/s41540-017-0023-2)
Supplement: Supplementary file 1 — Supplementary Methods 1 [file 41540_2017_23_MOESM1_ESM.docx]

**Performance of objective functions and optimisation procedures for parameter estimation in system biology models**

**Supplementary Methods 1**

Andrea Degasperi^1,4,a,c^, Dirk Fey^1,3,a^ and Boris N. Kholodenko^1,2,3^

1 Systems Biology Ireland, University College Dublin, Belfield, Dublin, Republic of Ireland

2 Conway Institute of Biomolecular & Biomedical Research, University College Dublin, Belfield, Dublin, Republic of Ireland

3 School of Medicine and Medical Science, University College Dublin, Belfield, Dublin, Republic of Ireland

4 Wellcome Trust Sanger Institute, Wellcome Trust Genome Campus, Hinxton, Cambridge, UK

a Equal contribution

c Corresponding author, e-mail address: andrea.degasperi@sanger.ac.uk

**Index**

1. Table of Notations
2. Objective functions for parameter estimation in dynamic modelling of signalling pathways using relative data
3. Issues with counting function evaluations, termination criteria and time-to-convergence
4. Acceptable parameter estimates can be reached long before the algorithms terminate
5. Estimation of the scaling factors before optimisation
6. Details of GLSDC and LevMar optimisation algorithms
7. **Table of Notations**

| $\tilde{y}_{kij}$ | Data point of observable $k\in K$, measured in condition (or time point) $i\in I$ in the biological replicate $j\in J$. Biological replicates are normalised, i.e. made comparable across the index $j$. |
| --- | --- |
| $y_{ki}\left( \theta\right)$ | Simulated concentration of observable $k\in K$ in condition (or time point) $i\in I$, using parameter set $\theta$. |
| $\alpha_{k}$ | Scaling factor that converts the absolute units of $y_{ki}\left( \theta\right)$ to the relative units of$\tilde{y}_{kij}$. A different scaling factor is assumed for each observable $k\in K$. |

1. **Objective functions for parameter estimation in dynamic modelling of signalling pathways using relative data**

Relative data are the most common type of experimental data (e.g., Western blotting^1,2^, multiplexed Elisa^3^, proteomics or RT-qPCR^4^). These data are expressed in arbitrary units, which are proportional to the amounts of protein, protein activities or mRNA levels. To allow data comparisons between biological replicates, different normalisation strategies can be used. For instance, the data obtained in each replicate are divided by the value of a reference point in that replicate, thus expressing fold change with respect to that point^5^.

Using such relative measurement data for parameter estimation requires matching the data units with the model units. For example, if the model is simulated in absolute units (e.g. nM), the simulated values have to be scaled (or normalised) to the relative units of the data. Similarly, even if the model is dimensionless, scaling (or re-normalisation) is still required, because it is unknown how the normalisation factor in the model relates to the data units. Matching data and model units can either be achieved by introducing scaling factors (SF), or by data-driven normalisation of the simulations (DNS). Scaling factors are additional unknown parameters that relate the units of the simulations to the measurement units. Obviously, these unknown scaling-factors have to be estimated, thus increasing the dimensionality of the parameter estimation problem. DNS avoids this problem by normalising the simulated and experimental data in exactly the same way. For example, if the data are biological replicates of Western blot intensities of a time course of phosphorylated ERK (pERK), these replicates may be from different blots and then normalised by the average of the intensities of each blot. The resulting relative units can be compared to the model simulation by multiplying the simulation by a scaling factor, which needs to be estimated, or by normalising the simulation by the average of the simulated values obtained for the same time points of the data, thus normalising the simulations and the data in the same way.

Another important choice in parameter estimation is the type of function to use as objective function. Three approaches are commonly used: the sum of squared differences, or least squares, (LS), the chi-squared function (Χ^2^), and the negative log likelihood (LL). Both the chi-squared function and the negative log likelihood require knowledge of the measurement error of each data point, which are used as weights for the squared differences: a larger noise corresponds to a lower weight, indicating that the corresponding squared difference has a lower impact in the overall objective function. In other words, a larger uncertainty of a data point allows a looser fit of the simulation to that data point. The log likelihood and the chi-squared approach coincide in the case where the measurement error is calculated from the data before optimisation. The measurement error is usually empirically obtained by computing the variance of each data point across replicates. Because of the typically low number of replicates available in biological experiments, often not more than three or four, this method of estimating the measurement error may be unreliable and sometimes not possible, e.g. when there are no replicates. Thus, we prefer to follow the approach of Ref. 6 and estimate the measurement error during the optimisation as a linear model. Details are given below, where we describe the objective functions formally. Here we call this method negative log likelihood with linear model of measurement error, or simply log likelihood (LL).

Finally, we describe formally the four objective functions we consider in this study: least squares with data-driven normalisation of the simulations (LS DNS), least squares with scaling factors (LS SF), log likelihood with data-driven normalisation of the simulations (LL DNS) and log likelihood with scaling factors (LL SF).

We assume that the variable $\tilde{y}$ represents the relative amount of observed biochemical species or species activity (where relative means that this quantity is measured in arbitrary units). We use variable $\tilde{y}_{kij}$ to indicate the data point of observable $k\in K$, measured in condition $i\in I$ in the biological replicate $j\in J$. In general, when data are collected, the data points are comparable across the $i$ index (e.g. different conditions or time points quantified from the same western blot) but are not comparable across biological replicates (index $j$)^5^. In the following we will assume that the data points $\tilde{y}_{kij}$ have been normalised either by the average of the data points in a biological replicate or by a fixed data point in a replicate, thus making the data points comparable across the index $j$.

Denoting with $\hat{y}_{kij}$ the data points not yet normalised, data normalisation can be defined as follows^5^.

- *Normalisation by fixed point.* After choosing a condition or time point $p\in I$ as the fixed point, the normalised data points $\tilde{y}_{kij}$ are defined as:

| $\tilde{y}_{kij}=\frac{\hat{y}_{kij}}{\hat{y}_{kpj}} \forall k\in K,i\in I,j\in J$ | (1) |
| --- | --- |

Notice that the condition or time point $p$ can be different for each observable $k\in K$. Data points $\tilde{y}_{kij}$ are now expressed as a fold change with respect to the fixed data point $\tilde{y}_{kpj}$.

- *Normalisation by average.* After choosing the set of conditions or time points $W\subseteq I$, with the size of the set $W$ denoted by $m$, the normalised data points $\tilde{y}_{kij}$ are defined as:

| $\tilde{y}_{kij}=\frac{\hat{y}_{kij}\cdot m}{\sum_{w\in W} \hat{y}_{kwj}} \forall k\in K,i\in I,j\in J$ | (2) |
| --- | --- |

Notice that the set $W$ can be different for each observable $k\in K$. The set $W$ may contain all conditions in $I$ ($W=I$) or a subset of them ($W\subseteq I$), such as in the case of missing values, or a subset of conditions where the experiments are particularly reliable and reproducible. A possible choice for the set of indexes $W$ is the largest set of indexes $w\in I$ such that $\hat{y}_{kwj}$ is available in all replicates $j$ of observable $k$.

An ODE system for modelling signalling pathways can be defined by the following equations:

$$\frac{dx\left( t \right)}{dt}=f\left( x\left( t \right),u\left( t \right),\theta\right)$$

$$y\left( t,\theta\right)=g\left( x\left( t \right),\theta\right)$$

In the equations above, $x\left( t \right)$ is a vector containing the state variables that are simulated, such as biochemical species and their level of activity. The variable $u\left( t \right)$ is a vector containing the input functions for the model, for example the concentration of a growth factor. The variable $\theta$ is a vector containing the parameters of the model. Finally, $y\left( t,\theta\right)$ represents the time-dependent observable. It is the output of the model that corresponds to the measured biochemical species or species activity. The observable $y\left( t,\theta\right)$ is a function of the model variables (usually their linear combination). For example, the observable of phosphorylated ERK may be the sum of all the model variables that represent complexes containing phosphorylated ERK. We use the variables $y_{ki}\left( \theta\right)$ to indicate the simulated value of the observable $k$ obtained with parameters $\theta$ and that corresponds to the data points $\tilde{y}_{kij}$ ($j\in J$, biological replicates).

**Least squares with scaling factors (LS SF)**. The objective function is as follows:

$$obj\left( \theta\right)=\sum_{k\in K} \sum_{i\in I} \sum_{j\in J} \left( y_{ki}\left( \theta\right)\cdot\alpha_{k}-\tilde{y}_{kij} \right)^{2}$$

In the above equation, $\alpha_{k}$ are the scaling factors that need to be estimated ($\alpha_{k}>0$). In general, one needs to estimate one scaling factor for each observable. Data points are normalised using either Equation 1 or Equation 2.

**Least squares with normalisation of the simulations (LS DNS).** We illustrate here two versions of this objective function: one when the data are normalised with respect to a fixed data point, and one when the data are normalised with respect to the average of the data points. In the first case, the data points $\hat{y}_{kij}$ are normalised with respect to a fixed data point $\hat{y}_{kpj}$, with $p\in I$, as shown in Equation 1, thus obtaining normalised data $\tilde{y}_{kij}$. This implies that the simulated data points $y_{ki}\left( \theta\right)$ need to be normalised with respect the simulated data point $y_{kp}\left( \theta\right)$:

$$obj\left( \theta\right)=\sum_{k\in K} \sum_{i\in I} \sum_{j\in J} \left( \frac{y_{ki}\left( \theta\right)}{y_{kp}\left( \theta\right)}-\tilde{y}_{kij} \right)^{2}$$

In the second case, the data points $\hat{y}_{kij}$ are normalised with respect to the average $\left( \sum_{w\in W} \hat{y}_{kwj} \right)/m$ , with $W\subseteq I$, and $m$ is the size of $W$. This normalisation is shown in Equation 2 and defines the normalised data points $\tilde{y}_{kij}$. Finally, this normalisation implies that simulated data points $y_{ki}\left( \theta\right)$ need to be normalised with respect to the corresponding average $\left( \sum_{w\in W} y_{kw}\left( \theta\right) \right)/m$:

$$obj\left( \theta\right)=\sum_{k\in K} \sum_{i\in I} \sum_{j\in J} \left( \frac{y_{ki}\left( \theta\right)\cdot m}{\sum_{w\in W} y_{kw}\left( \theta\right)}-\tilde{y}_{kij} \right)^{2}$$

**Log likelihood with scaling factors (LL SF).** As mentioned above, we consider the log likelihood where the measurement error is estimated as a linear model^6^. When estimating scaling factors the objective function is:

$$obj\left( \theta\right)=\sum_{k\in K} \sum_{i\in I} \sum_{j\in J} \left( \frac{y_{ki}\left( \theta\right)\cdot\alpha_{k}-\tilde{y}_{kij}}{\sigma_{ki}} \right)^{2}+2\sum_{k\in K} \sum_{i\in I} \sum_{j\in J} ln\left( \sigma_{ki} \right)$$

The measurement error is given by the linear model $\sigma_{ki}=s_{a}+s_{b}\cdot y_{ki}\left( \theta\right)\cdot\alpha_{k}$. The coefficients $s_{a}>0$ and $s_{b}>0$ are the coefficients of the linear model of the measurement error and need to be estimated. We assume $y_{ki}\left( \theta\right)\geq0$, which implies $\sigma_{ki}>0$. Data points are normalised using either Equation 1 or Equation 2.

**Log likelihood with normalisation of the simulations (LL DNS).** Similarly, as for the least squares case, we will consider two objective functions, one for the normalisation by fixed point and one for the normalisation by average. In the case of normalisation by fixed point we have:

$$obj\left( \theta\right)=\sum_{k\in K} \sum_{i\in I} \sum_{j\in J} \left( \frac{{y_{ki}\left( \theta\right)}/{y_{kp}\left( \theta\right)}-\tilde{y}_{kij}}{\sigma_{ki}} \right)^{2}+2\sum_{k\in K} \sum_{i\in I} \sum_{j\in J} ln\left( \sigma_{ki} \right)$$

With measurement error $\sigma_{ki}=s_{a}+s_{b}\cdot{y_{ki}\left( \theta\right)}/{y_{kp}\left( \theta\right)}$. In the case of normalisation by average we have:

$$obj\left( \theta\right)=\sum_{k\in K} \sum_{i\in I} \sum_{j\in J} \left( \frac{{y_{ki}\left( \theta\right)\cdot m}/{\sum_{w\in W} y_{kw}\left( \theta\right)}-\tilde{y}_{kij}}{\sigma_{ki}} \right)^{2}+2\sum_{k\in K} \sum_{i\in I} \sum_{j\in J} ln\left( \sigma_{ki} \right)$$

With measurement error $\sigma_{ki}=s_{a}+s_{b}\cdot{y_{ki}\left( \theta\right)\cdot m}/{\sum_{w\in W} y_{kw}\left( \theta\right)}$.

1. **Issues with counting function evaluations, termination criteria and time-to-convergence**

Methods to assess the practical performance of optimisation algorithms employ two basic units of measure: computation time and number of function evaluations. In this section, we discuss details and issues concerning their use.

*Computation time.* Performance can be compared directly by measuring how much computation time is necessary to reach a certain objective function minimum, or by analysing the convergence of the optimised value as a function of the computation time. Better algorithms reach lower values quicker. Note that CPUs with the same computing power must be used.

*Counting function evaluations*. If CPUs with different computing power are used, the performance of algorithms may still be compared counting how many function evaluations are required to reach a certain objective function minimum, or by analysing the convergence of the optimised value over the number of function evaluations. Better algorithms require fewer function evaluations. Note that counting function evaluations assumes that the objective function carries the same computational cost between algorithms, and further, that evaluating the objective function is the computationally most expensive part of the algorithms. Depending on the algorithms, these assumptions might be wrong (e.g. SE vs FD).

*Algorithm termination criteria*. The considered algorithms terminate for different reasons. LevMar terminates when all Latin-hypercube restarts have terminated (here 200). Hereby, each restart terminates when either of the following is triggered: i) the gradient becomes small, indicating that a minimum was reached, ii) a small objective function value is reached, iii) the maximum number of iterations is reached, iv) a numerical error during the ODE integration using the CVODE library is encountered. GLSDC terminates either when all individuals in the genetic algorithm population converge to the same parameter values, or when the maximum number of genetic algorithm generations is reached. (See section “Details of GLSDC and LevMar optimisation algorithms” below for more details).

*Termination time and function evaluations to terminate*. We use the term “termination time” to indicate the amount of computation time required for the optimisation to terminate and the term “function evaluations to terminate” to indicate the number of function evaluations required for the optimisation to terminate. Note that having terminated does not necessarily imply that an optimal parameter estimate has been found (see previous paragraph).

*Optimal parameter sets are often not unique.* Given a limited amount of experimental data, biological systems are often not identifiable. Non-identifiably means that not only one, but several optimal parameter sets exist that fit the data equally well. Therefore, it is good practice to repeat the optimisation multiple times^7^ using different initial guesses (for deterministic algorithms) and different realisations of the random number generator (for global, sampling-based searches). The resulting distribution of the parameter estimates can be used to analyse practical identifiability, and determine which parameters (or parameter relations) can be estimated uniquely^8,9^.

*Comparing optimal parameter sets.* A slightly lower objective function value does not necessarily imply a better parameter estimate. In some cases, minor differences in objective function values are not relevant from a practical, biological point of view. If the simulated trajectories already capture the overall shape of the recorded time- or dose-response data, and the final goodness-of-fit value depends on how the errors are distributed along the data-points, then the risk of overfitting is high, and these minor differences should be neglected. In practice, different parameter estimates may all be biologically acceptable. A good example is provided in Ref. 10, where several distinct parameter estimates produced similarly good fits to validation data.

*Comparing least-squares and log-likelihood objective functions*. The values of different objective functions cannot be compared directly, which raises the question of how we can compare the goodness-of-fit between least-squares and log-likelihood estimates. Our approach is to record the least-square values also in the log-likelihood setting. The computational cost of this operation is negligible, because in our algorithms computing the least-squares function is part of computing the log-likelihood.

*Comparing termination times between different algorithms may constitute an unfair comparison.* Different algorithms may use different convergence and termination criteria, which make an objective comparison of the termination times difficult. In practice, algorithms may terminate reaching very different objective function minima, or terminate long after an acceptable minimum was reached, thus making the termination time alone an insufficient measure of performance. Here, LevMar SE and LevMar SD can be compared directly, because the same termination criteria are used. In contrast, comparing termination times between LevMar and GLSDC is inappropriate, because of the different termination criteria used. The actual termination times critically depend on algorithm-specific, internal tuning parameters, such as the Latin-hypercube grid-size in LevMar, or the number of generations in GLSDC. For example, LevMar runs longer when the number of Latin hypercube restarts is increased. Similarly, GLSDC runs longer when the number of maximal allowable genetic algorithm generations is increased.

*Comparing the objective function convergence over computation time eliminates the issues arising from different termination criteria.* In general, one algorithm might get very close to the minima very early on, but not terminate until much later, whereas another algorithm might stay far away from the minima for long periods but terminate as soon as it comes close. Thus, to compare the different algorithms objectively, we analyse the *convergence curve* of the algorithms by plotting the optimised objective function value (y-axis) over either computation time or the number of function evaluations (Figure 3 of main text). To summarise this analysis, one can select specific time points on this convergence curve and compare the corresponding objective function values between the different objective functions and algorithms. The chosen time points should be points of particular interest on the convergence curve, for example representing when the fastest converging algorithm has reached acceptable minima. Here, these interesting points were 3 and 8 minutes for the test problem STYX-1-10 (Figure 4A,B of main text), 10 minutes and 2h for EGF/HRG-8-10 (Figure 4C,D of main text), and 24h for EGF/HRG-8-74 (Figure 4E of main text).

1. **Acceptable parameter estimates can be reached long before the algorithms terminate**

Here we investigate how quickly acceptable solutions are found, which might happen long before the algorithms terminate. To that end, we analysed the convergence curves of LevMar FD and LevMar SE for the STYX-1-10 and EGF/HRG-8-10 problems, for which acceptable values of the objective function lie below 1.9 and 3, respectively. These cut-offs have been chosen, because they are close to the global minima of 1.7 and 2, respectively (Figures 2A,B). Analysing the convergence (Figures 4A-D and Figures 5A-D), revealed that acceptable objective function values were reached by most of the optimisation runs after eight minutes for STYX-1-10, and after 120 minutes for EGF/HRG-8-10. Yet, the algorithms continued to optimize, not terminating until long after these times (Figures 3B,D). Thus, these termination times are not an accurate reflection of the convergence times, at least in practical terms.

The finding also implies that an analysis of the full convergence curve as a function of computation time is necessary for i) accurate performance comparisons between different optimisation algorithms, and ii) problem-specific fine-tuning of the termination criteria of each optimisation algorithm such that only the computation time necessary to reach the minima, or little more, is used. Although such fine-tuning is impractical when the only objective is to find a good parameter estimate, it might be worth the effort when performing several 100s or 1000s of estimation runs in order to characterise the acceptable parameter space and analysing practical identifiability^7^.

1. **Estimation of the scaling factors before optimisation**

Another objective function not investigated here has been proposed in Ref. 11, where the authors minimise the objective function with respect to the scaling factors analytically before the numerical optimisation of the objective function. This results in an expression for the scaling factors which depends on a combination of simulated values and data points and that is optimal with respect to the objective function to be estimated. In this section, we provide the formulation of the objective used in Ref. 11 using our notation. We also adapt such approach to the objective functions considered here.

In Ref. 11 the objective function of choice is the negative log likelihood with known measurement error:

$$obj\left( \theta\right)=\frac{1}{2}\sum_{k\in K} \sum_{i\in I} \sum_{j\in J} \left( \frac{y_{ki}\left( \theta\right)\cdot\alpha_{k}-\tilde{y}_{kij}}{\sigma_{ki}} \right)^{2}$$

In the equation above, the measurement error $\sigma_{ki}$ is considered known and constant, thus estimated from the data before optimisation. One can easily show that the optimal $a_{k}$ are given by:

$$\alpha_{k}=\frac{\sum_{i\in I} \sum_{j\in J} \frac{y_{ki}\left( \theta\right)\cdot\tilde{y}_{kij}}{\sigma_{ki}^{2}}}{\left| J \right|\cdot\sum_{i\in I} \frac{\left( y_{ki}\left( \theta\right) \right)^{2}}{\sigma_{ki}^{2}}} \forall k\in K$$

In the equation above, $J$ is the set of indexes of the biological replicates, while $\left| J \right|$, the size of $J$, is the number of biological replicates. The equation assumes that the data points $\tilde{y}_{kij}$ have been normalised using either Equation 1 or Equation 2, and thus are comparable across the index $j$. In this case, $\sigma_{ki}^{2}$ is fixed a priori.

For completeness, we provide the equation for the scaling factors optimised in this way in the case of least squares and negative log likelihood with linear model of noise. When using least squares, the scaling factor $\alpha_{k}$ is equal to:

$$\alpha_{k}=\frac{\sum_{i\in I} \sum_{j\in J} y_{ki}\left( \theta\right)\cdot\tilde{y}_{kij}}{\left| J \right|\cdot\sum_{i\in I} \left( y_{ki}\left( \theta\right) \right)^{2}} \forall k\in K$$

When using negative log likelihood with linear model of noise, there is no simple equation for the scaling factor $\alpha_{k}$, because of the dependency of the linear model on the scaling factor, $\sigma_{ki}=s_{a}+s_{b}\cdot y_{ki}\left( \theta\right)\cdot\alpha_{k}$.

1. **Details of GLSDC and LevMar optimisation algorithms**

**GLSDC (Genetic Local Search with Distance Independent Diversity Control)**


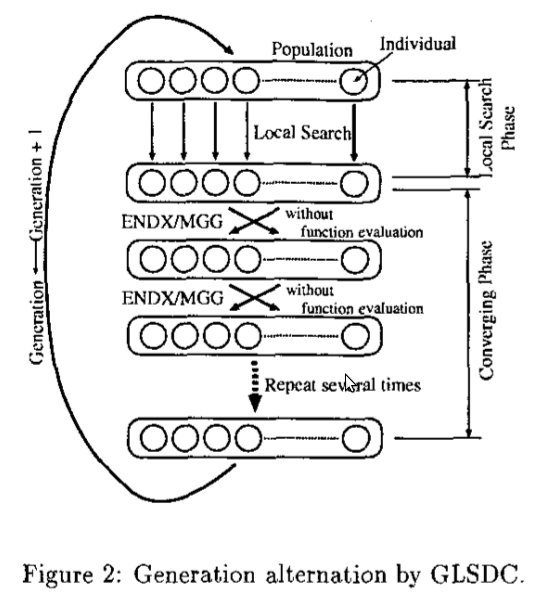


GLSDC alternates a local search phase based on the Powell’s method with a global converging phase based on a genetic algorithm. The algorithm can be described as follows:

1. Initialise np individuals randomly (np = number of parents). Each individual is defined by a set of parameter values. Set the number of iterations of the converging phase (see below), Niter = Nzero = np*convergingRatio.
2. **Repeat** at most MaxGenerations times alternating a Local phase and a convergence phase:
   1. **Local Search.** Apply the Powell’s method to each parent independently. Powell’s method is a local search algorithm that improves locally the parameter estimates of each individual, without explicit calculation of the gradient. Powell’s method iteratively performs linear searches towards a number of orthogonal directions in the unknown parameters space (with D dimensions). Two parameters are necessary: the number of iterations of the Powell’s method (Npowell) and the number of iterations of the single linear searches (Nlin). Powell’s method stops before Npowell iterations have been reached if 2*abs(best_old – best_new)/(abs(best_old) + abs(best_new)) is less than or equal to the parameter PowellTolerance.
   2. If the best objective function value from the previous generation has not improved, then set Niter = Niter + Nzero, otherwise set Niter = Nzero
   3. **Converging phase:** the ENDX/MGG genetic algorithm is used Niter times, without function evaluation. This phase is meant to make the values of the individuals converge towards the same values. At the same time it combines the individual parameter sets, making the algorithm a global search. Details of the ENDX/MGG algorithm:
      1. ENDX (Extended Normal Distribution Crossover). Select two random individuals from the population of np parents. Generate nc children according to the ENDX strategy (additional parameters alpha and beta required).
      2. MGG (Minimal Generation Gap): Choose two individuals in the family composed of two parents and their nc children. The two parents are replaced by a) the individual (parameter set) with the best objective function value in the family and b) a random individual in the family. In GLSDC, this phase is without function evaluation, which means that the objective function value of the children is not computed and assumed worse (higher value in minimisation) than the value of the parents. So, in practice, one random parent is kept and another random member of the family (more likely one of the nc children, but could be the other parent). The objective function value of a newly selected children will be evaluated at the next Local Search Phase.
   4. Stop if the halting criteria is satisfied. Otherwise go to a. Halting criteria: stop if for all dimensions (unknown parameters) the difference between the population’s largest parameter value and the population’s lowest parameter value is less than or equal to the parameter GLSDC_threshold.

Additional complication may arise when a function evaluation fails. This is usually due to numerical approximation errors in the CVODE library integration of the Ordinary Differential Equations (ODE) that need to be solved to calculate the objective function. For example, a variable of the ODE model could become negative by an absolute amount greater than the absolute tolerance parameter. If the function evaluation fails, then GLSDC is often able to recover, by bringing forward members of the population that evaluated correctly.

Parameters used:

- Maximum number of generations: 50
- Number of individuals (also called number of parents, np): if the dimensions D (number of unknown parameters) are less than 10, then np = 2 + D, otherwise np = 12
- Maximum iterations of the Powell’s method: Npowell = 10
- Converging ratio: 2.0
- ENDX: alpha=0.434, beta=0.35/sqrt(m-3), with m=np.
- GLSDC threshold for parameter convergence in the population: GLSDC_threshold = 10^-6
- Maximum iterations of the linear search used by the Powell’s method: Nlin = 10
- Number of children (nc) in the converging phase: 10
- Powell’s method convergence parameter: PowellTolerance = 10^-5

**LevMar (Levenberg-Marquardt local non-linear least squares algorithm)**

LevMar can be thought of as a combination of steepest descent and the Gauss-Newton method. The combination of the two mentioned gradient based algorithms is weighted by the parameter mu, called the damping factor. When mu is large, LevMar behaves as the steepest descent method, which is usually more appropriate far from the local minimum. When mu is small, LevMar behaves as the Gauss-Newton method, which is usually more appropriate close to the local minimum. At each iteration, the gradient is computed and movement in the direction of the gradient is attempted. Several attempts can be made using the different mu, until an improved objective function value is obtained.

In order to use this local search algorithm for non-linear global optimisation, a restart approach is used. The algorithm is started Nrestarts times using different random initial points. We employ latin hypercube restarts to guarantee that the initial points are spread across the search space and are not by chance next to each other. Latin hypercube divides each dimension in Nrestarts segments. These segments define Nrestarts^D (D=number of dimensions) hypercubes. At each restart a random initial point is uniformly sampled within one of the hypercubes. The hypercube is selected randomly, ensuring that a certain segment of a certain dimension is not picked twice.

Variables:

- The unknown parameters vector p (p has dimension D)
- The error vector that contains the signed difference between the data and the function value e=(x-f(p)).
- The gradient or Jacobian matrix J = de/dp

Terminating criteria for each of the Nrestarts local optimisations:

1. Small gradient: $\left\| J^{T}e \right\|_{Inf}\leq TOL$
2. Small p: $\left\| p \right\|_{2}\leq TOL$
3. Small error e: $\left\| e \right\|_{2}\leq TOL$
4. Iteration limit Niter
5. Mu cannot be increased further (numerical overflow)
6. Invalid (NaN or Inf) function value or gradient. This error may happen because of numerical errors in the CVODE library during ODE integration.

Parameters used:

- Number of latin hypercube restarts: Nrestarts = 200
- Number of maximum iterations: Niter = 100
- Delta for the forward finite difference approximation of the gradient: delta = max(10^-4*abs(p),10^-6). In words delta is whatever is larger between 10^-6 and the absolute value of the parameter p multiplied by 10^-4. The parameter p is the current value of the parameter for which the gradient is computed.
- Tolerance for small value: TOL=10^-17

**References**

1 Burnette, W. N. "Western blotting": electrophoretic transfer of proteins from sodium dodecyl sulfate--polyacrylamide gels to unmodified nitrocellulose and radiographic detection with antibody and radioiodinated protein A. *Analytical biochemistry* **112**, 195-203 (1981).

2 Towbin, H., Staehelin, T. & Gordon, J. Electrophoretic transfer of proteins from polyacrylamide gels to nitrocellulose sheets: procedure and some applications. *Proceedings of the National Academy of Sciences of the United States of America* **76**, 4350-4354 (1979).

3 Tighe, P. J., Ryder, R. R., Todd, I. & Fairclough, L. C. ELISA in the multiplex era: potentials and pitfalls. *Proteomics Clin Appl* **9**, 406-422, doi:10.1002/prca.201400130 (2015).

4 Heid, C. A., Stevens, J., Livak, K. J. & Williams, P. M. Real time quantitative PCR. *Genome Res* **6**, 986-994 (1996).

5 Degasperi, A. *et al.* Evaluating strategies to normalise biological replicates of Western blot data. *PloS one* **9**, e87293, doi:10.1371/journal.pone.0087293 (2014).

6 Raue, A. *et al.* Lessons learned from quantitative dynamical modeling in systems biology. *PloS one* **8**, e74335, doi:10.1371/journal.pone.0074335 (2013).

7 Hengl, S., Kreutz, C., Timmer, J. & Maiwald, T. Data-based identifiability analysis of non-linear dynamical models. *Bioinformatics* **23**, 2612-2618, doi:10.1093/bioinformatics/btm382 (2007).

8 Gutenkunst, R. N. *et al.* Universally sloppy parameter sensitivities in systems biology models. *Plos Comput Biol* **3**, 1871-1878, doi:10.1371/journal.pcbi.0030189 (2007).

9 Transtrum, M. K. *et al.* Perspective: Sloppiness and emergent theories in physics, biology, and beyond. *J Chem Phys* **143**, 010901, doi:10.1063/1.4923066 (2015).

10 Reiterer, V., Fey, D., Kolch, W., Kholodenko, B. N. & Farhan, H. Pseudophosphatase STYX modulates cell-fate decisions and cell migration by spatiotemporal regulation of ERK1/2. *Proceedings of the National Academy of Sciences of the United States of America* **110**, E2934-2943, doi:10.1073/pnas.1301985110 (2013).

11 Weber, P., Hasenauer, J., Allgöwer, F. & Radde, N. Parameter Estimation and Identifiability of Biological Networks Using Relative Data. *IFAC Proceedings Volumes* **44**, 11648-11653, doi:<http://dx.doi.org/10.3182/20110828-6-IT-1002.01007> (2011).
