# Supplementary figures and images for: Performance of objective functions and optimisation procedures for parameter estimation in system biology models
Source: NPJ Syst Biol Appl. 2017 Aug 8;3:20. doi: 10.1038/s41540-017-0023-2 (PMC5548920; doi:10.1038/s41540-017-0023-2)

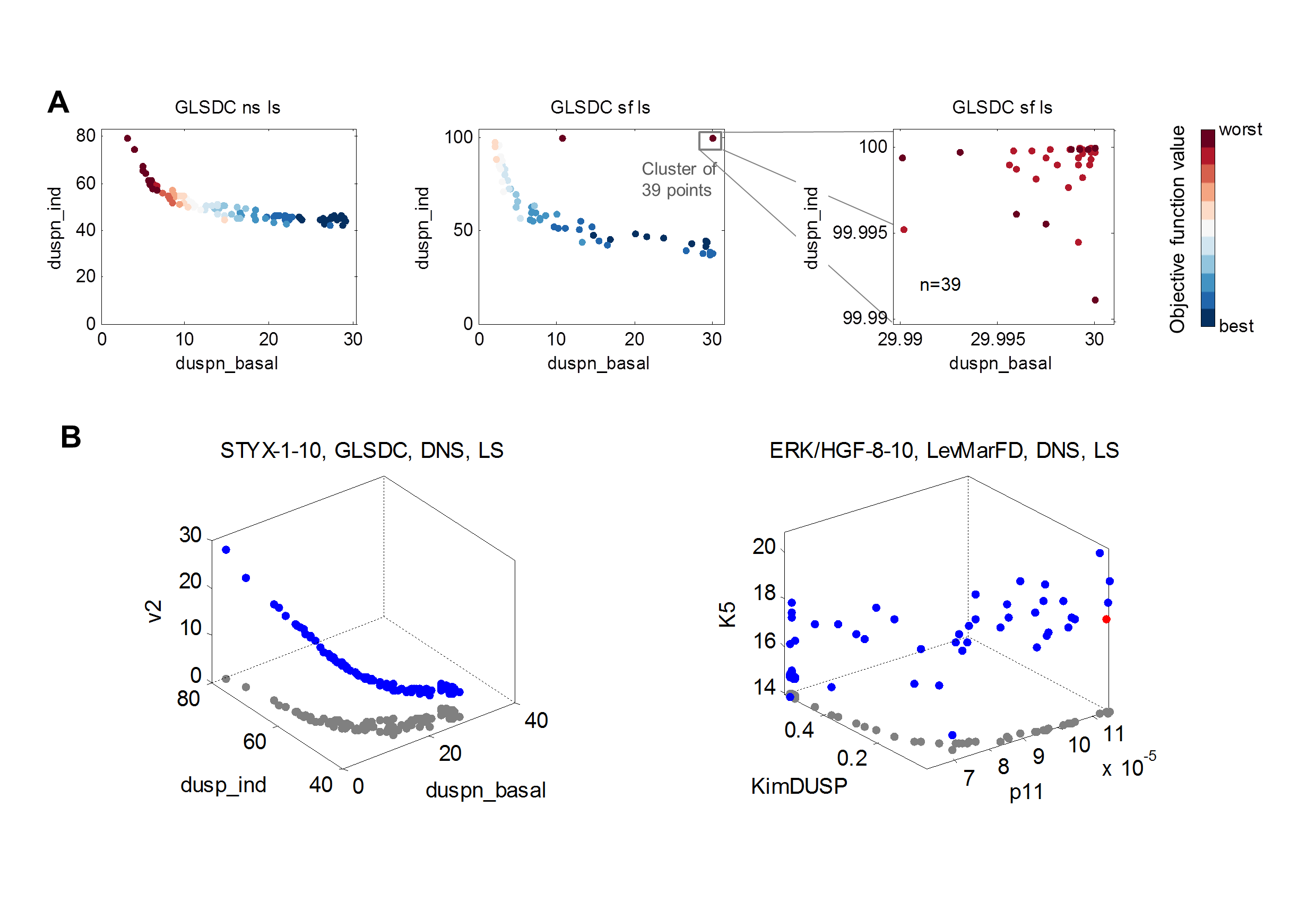

Supplement: Supplementary file 2 — Supplementary Figure 1 [file 41540_2017_23_MOESM2_ESM.tif]

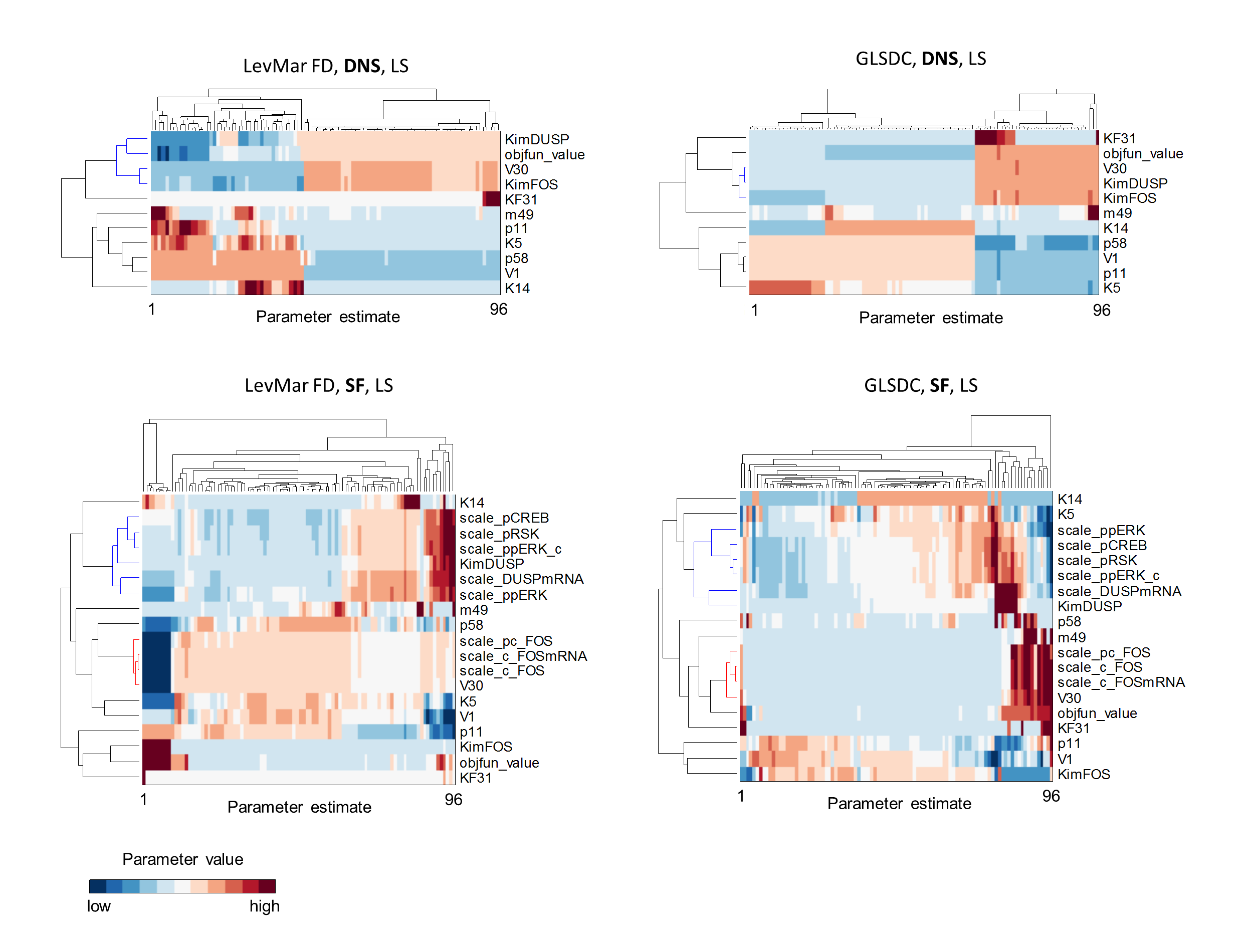

Supplement: Supplementary file 3 — Supplementary Figure 2 [file 41540_2017_23_MOESM3_ESM.tif]
